# Exploring the Equilibrium State Diagram of Maltodextrins across Diverse Dextrose Equivalents

**DOI:** 10.3390/polym16142014

**Published:** 2024-07-14

**Authors:** Zenaida Saavedra-Leos, Anthony Carrizales-Loera, Daniel Lardizábal-Gutiérrez, Laura Araceli López-Martínez, César Leyva-Porras

**Affiliations:** 1 Multidisciplinary Academic Unit, Altiplano Region (COARA), Autonomous University of San Luis Potosi, Carretera a Cedral km 5+600, Matehuala 78700, Mexico; zenaida.saavedra@uaslp.mx (Z.S.-L.); a275849@alumnos.uaslp.mx (A.C.-L.); 2 Advanced Materials Research Center (CIMAV), Miguel de Cervantes 120, Complejo Industrial Chihuahua, Chihuahua 31136, Mexico; daniel.lardizabal@cimav.edu.mx; 3 Academic Coordination of the Western High Plateau Region, Autonomous University of San Luis Potosi, Salinas de Hidalgo 78600, Mexico; araceli.lopez@uaslp.mx

**Keywords:** quercetin microencapsulation, maltodextrins, dextrose equivalents, equilibrium state diagram, storage conditions

## Abstract

This study investigates the equilibrium state diagram of maltodextrins with varying dextrose equivalents (DE 10 and 30) for quercetin microencapsulation. Using XRD, SEM, and optical microscopy, three transition regions were identified: amorphous (a_w_ 0.07–0.437), semicrystalline (a_w_ 0.437–0.739), and crystalline (a_w_ > 0.739). In the amorphous region, microparticles exhibit a spherical morphology and a fluffy, pale-yellow appearance, with Tg values ranging from 44 to −7 °C. The semicrystalline region shows low-intensity diffraction peaks, merged spherical particles, and agglomerated, intense yellow appearance, with Tg values below 2 °C. The crystalline region is characterized by fully collapsed microstructures and a continuous, solid material with intense yellow color. Optimal storage conditions are within the amorphous region at 25 °C, a_w_ 0.437, and a water content of 1.98 g H_2_O per g of dry powder. Strict moisture control is required at higher storage temperatures (up to 50 °C) to prevent microstructural changes. This research enhances understanding of maltodextrin behavior across diverse dextrose equivalents, aiding the development of stable microencapsulated products.

## 1. Introduction

Food polymers are polymeric materials that can be safely consumed by humans, animals, or microorganisms [1]. These materials are categorized into polysaccharides, proteins, and lipids. The use of food polymers has become increasingly significant in the development of functional food products, including food packaging and nutrient protection. Additionally, they are pivotal in biomedical applications such as controlled drug delivery, tissue engineering, and wound coatings [2,3]. Compared to synthetic polymers, food polymers offer notable advantages, including biodegradability, biocompatibility, and recyclability [4,5].

Currently, the growing interest in nutrition and health has driven the development of foods that not only meet basic dietary needs but also provide additional health benefits [6]. This trend has led to the creation of functional foods, which fulfill nutritional requirements while positively impacting one or more bodily functions. These foods can also play a preventive role by reducing risk factors associated with various diseases [7]. In addition to these health benefits, it is crucial that food products maintain their properties such as nutritional value, taste, texture, and safety under various external environmental conditions. The primary goal of food conservation is to prolong the shelf life of food, ensuring that it remains safe and enjoyable to consume over an extended period [8]. The main factors affecting shelf life are as follows: (i) the storage conditions, such as temperature, humidity, and light exposure; (ii) the type of packing for protecting the product from the environment; (iii) the stability and composition of the ingredients used in the product; (iv) processing methods used for reducing sources of degradation such as pasteurization, sterilization, and dehydration; (v) the use of chemical or natural preservatives can help prolong shelf life. By understanding and optimizing these factors, manufacturers can ensure that their products remain high-quality and safe for consumption over a longer period.

State diagrams are graphical representations that depict the different states of a substance or mixture under varying conditions of temperature, pressure, and composition [9]. In the context of food stability, these diagrams are essential for understanding and predicting the behavior and stability of food materials under different environmental conditions [10]. State diagrams typically include the following components: isotherms, temperature at which they were constructed, water activity, moisture content, and glass transition temperature [11]. By analyzing a state diagram, it is possible to determine the optimal moisture content and temperature for storing dehydrated products to prevent microbial growth [10]. Additionally, the diagram can reveal the conditions under which the product will transition from a glassy to a rubbery state, indicating potential risks for caking or loss of crispness [12]. Understanding the relationships between temperature, moisture content, and other factors through state diagrams allows for the development of more effective preservation, storage, and processing techniques. This ultimately leads to safer and longer-lasting food products.

Maltodextrins are polysaccharides derived from starch through a process called partial hydrolysis, which breaks down the starch into smaller carbohydrate molecules [13]. These molecules are composed of glucose units linked together in chains of varying lengths [14]. Their structure and properties are determined by the degree of polymerization (DP) and the extent of hydrolysis, reflected in the dextrose equivalent (DE). DE is a measure of the amount of reducing sugars present in a sugar product, expressed as a percentage on a dry basis relative to pure dextrose (glucose), which has a DE value of 100. It quantifies the degree of hydrolysis of starch into glucose and other reducing sugars. A higher DE value indicates a greater degree of starch hydrolysis, resulting in shorter chains of glucose units and higher sweetness. Maltodextrin refers to hydrolysates with DE values ranging from 3 to 20, whereas those with DE values above 20 are classified as glucose syrups. Due to their abundant hydroxyl (–OH) groups and branching structures, maltodextrins are highly soluble in water, making them easy to incorporate into different formulations [15,16]. In the food and beverage industry, maltodextrins serve multiple purposes. They enhance texture, act as bulking agents, and stabilize products [17]. Typically, they are white, odorless powders with a neutral to slightly sweet taste, depending on their degree of polymerization. Similar to other carbohydrates, maltodextrins provide about four calories per gram [18]. There is an empirical rule that inversely relates the DP to the DE (DP = 120/DE), indicating that as the DE increases, the DP decreases. For example, maltodextrins with DE values of 10 and 30 have estimated DP values of 12 and 4, respectively. Maltodextrin with a DE of 10 consists predominantly of long-chain oligosaccharides composed of glucose units, with largely linear chains and some degree of branching. In contrast, maltodextrin with a DE of 30 is composed of shorter chains of glucose units, resulting from more extensive hydrolysis, leading to a higher proportion of short oligosaccharides and even monosaccharides. The DE value impacts properties such as solubility, sweetness, and viscosity, with lower DE maltodextrins being less soluble, less sweet, and more viscous, while higher DE maltodextrins are more soluble, sweeter, and less viscous, making them suitable for different industrial applications. Overall, maltodextrins are versatile and valuable additives in various applications due to their solubility, neutral flavor, and functional properties.

Maltodextrins are widely used as microencapsulating agents in the food, pharmaceutical, and cosmetic industries [19,20,21]. Microencapsulation is a process where active ingredients or sensitive compounds are coated with a protective material to improve their stability, control release, and enhance their handling properties [22,23]. Their use in encapsulating flavors, vitamins, probiotics, colorants, pharmaceuticals, and cosmetic ingredients highlights their importance in improving product quality and functionality [24]. For example, Saavedra-Leos et al. [25] studied a set of four maltodextrin powders with varying DE and DP, all processed via spray drying. Their findings revealed a direct relationship between DE and DP, with the degree of polymerization emerging as a better parameter for describing the microstructure of maltodextrins. Later, Saavedra-Leos et al. [26] compared the performance of maltodextrin and inulin as microencapsulating agents for antioxidants contained in blueberry juice. The study reported that after spray drying, the maltodextrin powder retained a higher concentration of antioxidants, demonstrating superior performance in preserving these valuable compounds. In this context, it was found that for spray drying orange juice with maltodextrin as the carrier agent, the microstructure of the powders obtained using maltodextrins with a DE of less than 30 was stable and did not collapse. This was observed as a white, non-agglomerated powder [27]. Based on the DE, the type of maltodextrin plays a significant role in the yield and antioxidant content of microencapsulated products obtained through spray drying. Maltodextrins with a DE of less than 10 demonstrated higher content and yield values compared to those with DE of 20 and 40 for both quercetin and resveratrol [28,29]. Maltodextrins have been used as carrier agents in the drying of other juices, such as broccoli and strawberry juices, where the properties of these juices, including aroma, color, and composition, were preserved in the resulting dry powders [30,31]. Additionally, the effects of maltodextrin–inulin blends on the preservation of antioxidants and probiotics have been reported [32]. Recently, an equilibrium state diagram for a functional powdered food based on one of these mixtures (25% maltodextrin and 75% inulin) was documented [33]. From this diagram, optimal storage conditions to extend shelf life were identified. These conditions include a monolayer water content of 2.79 g of water per 100 g of dry powder and a storage temperature slightly above 30 °C. However, to the best of our knowledge, an equilibrium state diagram for maltodextrin has not been reported, nor has the effect of DE on this diagram been explored.

Therefore, the aim of this investigation is to develop a functional food with technological application that also provides antioxidant properties, utilizing spray drying as a microencapsulation technique. For the production of the functional powdered food, maltodextrins with varying degrees of dextrose equivalent (DE) were enriched with quercetin as the antioxidant agent. Equilibrium state diagrams for the microencapsulated product (quercetin/maltodextrin) will be employed to understand its behavior under specific environmental conditions (storage temperature and humidity) and to delineate the precise conditions essential for preserving food stability and ensuring the sustained efficacy of the antioxidant properties within the product.

## 2. Materials and Methods

### 2.1. Materials

Two commercial maltodextrins with different DE values, DE 10 and DE 30, were used (≥99% purity, Ingredion, Mexico city, Mexico). The antioxidant employed was quercetin (≥95% purity, Merck, Toluca, Mexico). The adsorption isotherms were developed using microenvironments with different relative humidity. This was achieved using the following inorganic salts: sodium hydroxide (NaOH), potassium acetate (CH_3_COOK), magnesium chloride (MgCl_2_), potassium carbonate (K_2_CO_3_), magnesium nitrate (Mg(NO_3_)_2_), sodium nitrate (NaNO_3_), potassium chloride (KCl), and potassium sulfate (K_2_SO_4_) (≥99% purity, Fermont, Monterrey, Mexico).

### 2.2. Methods

#### 2.2.1. Spray Drying

For the microencapsulation of the antioxidant, spray drying was employed. A 100 mL feed solution was prepared using 20 g of the respective carrier agent (Maltodextrin DE 10 or DE 30), 1 g of quercetin, and distilled water. The microencapsulation process was carried out using a Mini Spray Dryer B290 (BÜCHI, Labortechnik AG, Flawil, Switzerland) under the following conditions: a feed flow rate of 7 cm^3^/min, a hot air flow rate of 28 m^3^/h, 70% aspiration, and a pressure of 1.5 bar. Based on the findings reported by Saavedra-Leos et al. (2022), an inlet temperature of 210 °C was selected to achieve the highest antioxidant activity in the resulting product [32].

#### 2.2.2. Sorption Isotherms

Sorption isotherms were determined using the static gravimetric method as proposed by Labuza et al. [34]. The moisture content of the obtained products was measured using the oven-drying method [35]. Approximately 2 g of dried food product powder were placed into closed glass containers, comprising different saturated reagents to achieve the desired water activity (a_w_) at equilibrium. The reagents used and the corresponding a_w_ achieved were NaOH (0.070), CH_3_COOK (0.225), MgCl_2_ (0.329), K_2_CO_3_ (0.437), Mg(NO_3_)_2_ (0.531), KCl (0.739), NaCl (0.84), and K_2_SO_4_ (0.96). The incubation temperature was set at 25 °C, reflecting the typical room temperature in storage warehouses. Weights were recorded every 24 h until a constant weight was achieved, defined by a difference of ±0.001 g between two consecutive weightings. The isotherms were constructed based on the water activity data and the equilibrium moisture content of the evaluated samples. The mathematical models used to describe sorption behavior are presented in Table 1.

#### 2.2.3. Antioxidant Activity (AA)

The functional powdered food was evaluated for its hydrogen-donating or radical-scavenging activity using 2,2-Diphenyl-1-picrylhydrazyl (DPPH) as the standard. A mixture of 1.7 mL of an alcoholic DPPH solution (0.1 mmol DPPH) and 1.7 mL of a microencapsulated suspension at a concentration of 30 µg/mL was prepared. The mixture was left to stand in the dark for 30 min, and the absorbance was measured at 537 nm using a UV-Vis Evolution 220 spectrophotometer (Thermo Scientific, Waltham, MA, USA). Measurements were performed in triplicate after the spray drying process and during storage under different humidity conditions.

#### 2.2.4. Physicochemical Characterization

To characterize the microstructure of the functional food product, X-ray diffraction (XRD) analysis was performed. This was conducted using a D8 Advance ECO diffractometer (Bruker, Karlsruhe, Germany) equipped with Cu-Kα radiation (λ = 1.5406 Å), operating at 45 kV and 40 mA in Bragg–Brentano geometry. Scans were conducted over a 2θ range of 5° to 50°, with a step size of 0.016° and a scan time of 20 s per step.

Morphological analysis was carried out with a field emission scanning electron microscope (FESEM) (JEOL JSM-7401F, Tokyo, Japan) at an acceleration voltage of 2 kV. Powder samples were spread on double-sided conductive copper tape and coated with a thin gold layer using a sputter coater (Denton Desk II, Denton, TX, USA) to minimize charging effects. Images were captured at various magnifications: 500×, 1000×, 2500×, and 5000×.

The glass transition temperature (Tg) was determined following the procedure outlined by Saavedra-Leos et al. [36]. A Q200 modulated differential scanning calorimeter (MDSC) (TA Instruments, Lukens Drive, New Castle, DE, USA), equipped with an RCS90 cooling system, was utilized. The calorimeter was calibrated for temperature and enthalpy using indium, and for heat capacity using sapphire. Nitrogen gas of HPLC-grade purity was used as the purge gas at a flow rate of 100 mL/min. Approximately 10 mg of each sample was placed in sealed Tzero™ aluminum pans. The thermal program involved heating and cooling cycles with a modulation period of 40 s and an amplitude of 1.5 °C. The temperature range was set from −35 to 240 °C.

## 3. Results

### 3.1. Sorption Isotherms at 25 °C

**Figure 1:**
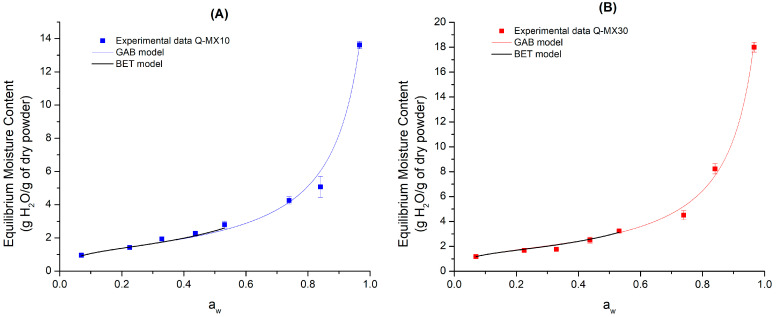
Adsorption isotherms for quercetin-maltodextrin functional food stored at 25 °C: (**A**) Q-MX10 (DE 10); (**B**) Q-MX30 (DE 30).

Figure 1A presents the adsorption isotherm for the quercetin–maltodextrin sample with a DE of 10 (Q-MX10) stored at 25 °C. The experimental equilibrium moisture content values (squared data points) for the functional food product were fitted using the GAB and BET mathematical models (solid lines).

The GAB model is described by the monolayer moisture content (Xm) and the constants C and K. The first parameter refers to the molecular layer that is bound to free sites which can be occupied by water, creating an aqueous phase in the food. The constants C and K, on the other hand, are related to the amount of energy required to remove water from the monolayer and multilayer, respectively [37]. The mathematical fitting of the GAB model indicates that the Q-MX10 sample exhibits three adsorption zones: (i) a steady increase in moisture content from 1.10 to 2.3 g H_2_O per g of dry powder within an a_w_ range of 0.07 to 0.53; (ii) an increase in moisture content from 2.37 to 5.76 g H_2_O per g of dry powder within an a_w_ range of 0.53 to 0.73; (iii) an accelerated adsorption from 5.76 to 13.53 g H_2_O per g of dry powder within an a_w_ range of 0.73 to 0.967. Regarding the model fitting parameters, the following values were obtained: Xm = 1.29, C = 27.55, K = 0.937, and R^2^ = 0.993. The GAB equation is one of the most widely used models for predicting the shelf life of food. It is based on the theory of monolayer and multilayer adsorption, where the food tends to absorb moisture from the environment through the multilayer and subsequently the monolayer. The results obtained in this research align with those reported by Navia et al. [38], who conducted a study on the development of a cassava flour and glycerol biopellicle. They determined the barrier properties of this product by performing sorption isotherms at 15, 25, and 35 °C. These researchers fitted the experimental data to the GAB, Oswin, Smith, and Henderson mathematical models. They reported that the GAB model provided the best fit at all three temperatures. Specifically, at 25 °C, the GAB model parameters were as follows: Xm = 4.52, C = 1.65, and K = 0.84 with an R^2^ value of 0.99.

The BET model, on the other hand, is characterized by the monolayer water content (Xm) and the constant C. However, the BET model has a limitation regarding the range of water activity in which it is valid, so the determination of adsorption phenomena was carried out up to an a_w_ value of 0.531. For the Q-MX10 sample, the parameters obtained from the BET model fit were as follows: Xm = 1.25, C = 28.31, and R^2^ = 0.941. Unlike the GAB model, the adsorption isotherm described by the BET model shows only two adsorption zones: (I) the first corresponds to a constant increase in moisture content from 0.9884 to 1.3836 g H_2_O per g of dry powder in an a_w_ range of 0.07–0.225; (II) the second shows a significant increase in equilibrium moisture content from 1.3836 to 2.8666 g H_2_O per g of dry powder in an a_w_ range of 0.225–0.531. These results are consistent with those reported by Talens Oliag et al. [39], who observed moisture adsorption behavior using the BET model for breakfast cereals stored at 20 °C, obtaining Xm = 0.0514 and C = 7.574. Similarly, Cerviño et al. [40] reported that the BET model provided the best fit for describing the stability of sweet potato candies at 30 °C. They also reported an isotherm with two adsorption intervals, where the best BET model fit was observed in the a_w_ range of 0.0–0.5.

Figure 1B displays the adsorption isotherm for sample Q-MX30 (squared data points) stored at 25 °C. The solid lines represent the fit of the GAB and BET mathematical models.

The GAB model fit revealed three zones of moisture absorption: (I) a constant increase in moisture content from 0.9948 to 3.0609 g H_2_O per g of dry powder, corresponding to an a_w_ range of 0.07–0.53; (II) a moisture increment from 3.0609 to 7.6387 g H_2_O per g of dry powder in an a_w_ range of 0.53–0.84; (III) an accelerated adsorption from 7.6387 to 18.0512 g H_2_O per g of dry powder in an a_w_ range of 0.84–0.967. The parameters obtained for the GAB model were as follows: Xm = 1.58, C = 31.48, K = 0.946, and R^2^ = 0.971. These results align with those reported by Domínguez-Domínguez et al. [41], who studied the adsorption isotherm behavior for hibiscus seed (*Hibiscus sabdariffa* L.) at 25, 35, and 45 °C. Specifically, at 25 °C, for the GAB model, they obtained R^2^ values of 0.9895, 0.9894, and 0.9872 for the Criolla, China, and Sudan varieties, respectively. According to their findings, the GAB model was the best fit for adsorption isotherms of low-moisture foods. Pascual-Pineda et al. [42], investigated the storage conditions of dehydrated foods using adsorption isotherms at temperatures of 15, 25, and 35 °C, analyzing three low-moisture powdered foods: sucrose–calcium, pineapple, and paprika. They reported that the GAB equation fits powdered products well, with R^2^ values exceeding 0.99. Brousse et al. [43] conducted a study on the stability of dehydrated cassava puree (*Manihot esculenta* Crantz) using adsorption isotherms at temperatures of 25, 35, and 45 °C. They concluded that both the GAB and BET mathematical models accurately fit the experimental data, with a relative mean error of less than 10%. Additionally, the primary difference in the results arises from the fact that the BET model accounts only for monolayer adsorption, while the GAB model considers the properties of water adsorbed in multilayers. The calculated BET parameter for sample Q-MX30 were Xm = 1.50, C = 35.62, and R^2^ = 0.974.

When comparing the fitting values of the BET and GAB models between the two types of maltodextrins (DE 10 versus DE 30), it is observed that the Q-MXDE30 sample exhibited a higher monolayer moisture content for both models. This suggests that maltodextrin with a higher DE (i.e., DE 30) adsorbs slightly more moisture. This can be explained based on the chemical structure of maltodextrins, which consist of a main carbohydrate chain (amylose) and branches (amylopectin). The arrangement of these components results in differences in dextrose equivalents (DE). According to Saavedra-Leos et al. [25], a higher DE value indicates a greater number of functional groups available for interaction with water molecules, as well as a higher percentage of branching.

### 3.2. Physicochemical Characterization

**Figure 2:**
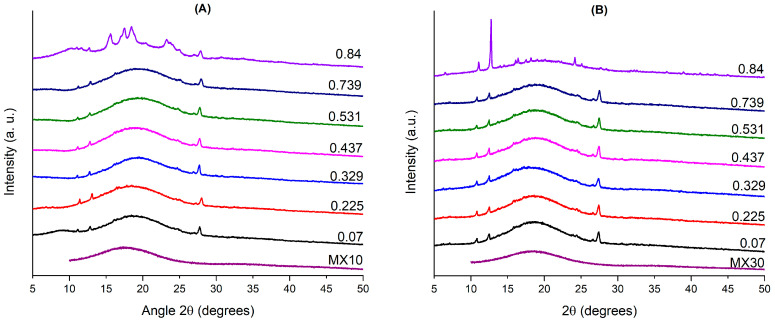
X-ray diffraction patterns of samples Q-MX10 (**A**) and Q-MX30 (**B**) under various moisture adsorption conditions at 25 °C.

Figure 2 presents the X-ray diffraction patterns of samples Q-MX10 and Q-MX30 after being subjected to different moisture adsorption conditions at 25 °C. Generally, the diffractograms exhibited similar behavior, featuring a broad peak around 18° and several low-intensity diffraction peaks at 11°, 12.5°, 16°, and 27°. The broad peak is characteristic of amorphous maltodextrin, while the low-intensity diffraction peaks are associated with the crystallization of quercetin [32,33]. However, for the Q-MX10 sample stored at an a_w_ of 0.840, there was a notable decrease in the intensity of the broad peak at 18° and the emergence of high-intensity diffraction peaks at 2θ angles of 16°, 17.5°, 18°, and 28°. In contrast, the Q-MX30 sample stored at the same a_w_ displayed different behavior, showing the appearance of a high-intensity peak at 17°, the disappearance of the broad peak at 18°, and the emergence of relatively low-intensity diffraction peaks between 15° and 20° and at 24°. This reduction in diffraction intensity at 18° is linked to the effect of absorbed water, which promotes crystallization. This suggests that the microstructure of both MD10 and MD30 undergoes changes in stability at high a_w_ values, transitioning from a glassy solid state to a crystalline solid state. Ballesteros et al. [44] conducted a comparative study on the encapsulation of antioxidant phenolic compounds extracted from coffee using both lyophilization and spray drying methods. Regarding the XRD analysis of samples using maltodextrin as a carrier agent, they found a very low degree of crystallinity. The samples exhibited a broad peak at 18° in 2θ, characteristic of such products, which is attributed to the amorphous nature of the powders obtained through spray drying. Matsuura et al. [45] conducted a study on the effect of dextrose equivalent in maltodextrin on the stability of coconut oil powder obtained through spray drying. The XRD results for all samples showed a characteristic diffraction at a 2θ angle of 20°, observed as a broad peak, which is indicative of amorphous materials. Zhang et al. [46] conducted a study on the use of maltodextrin as an encapsulating agent for xylooligosaccharides during the spray drying process. They concluded that using maltodextrin as a carrier material during spray drying promotes the formation of amorphous solid materials with high dissolution rates.

**Figure 3:**
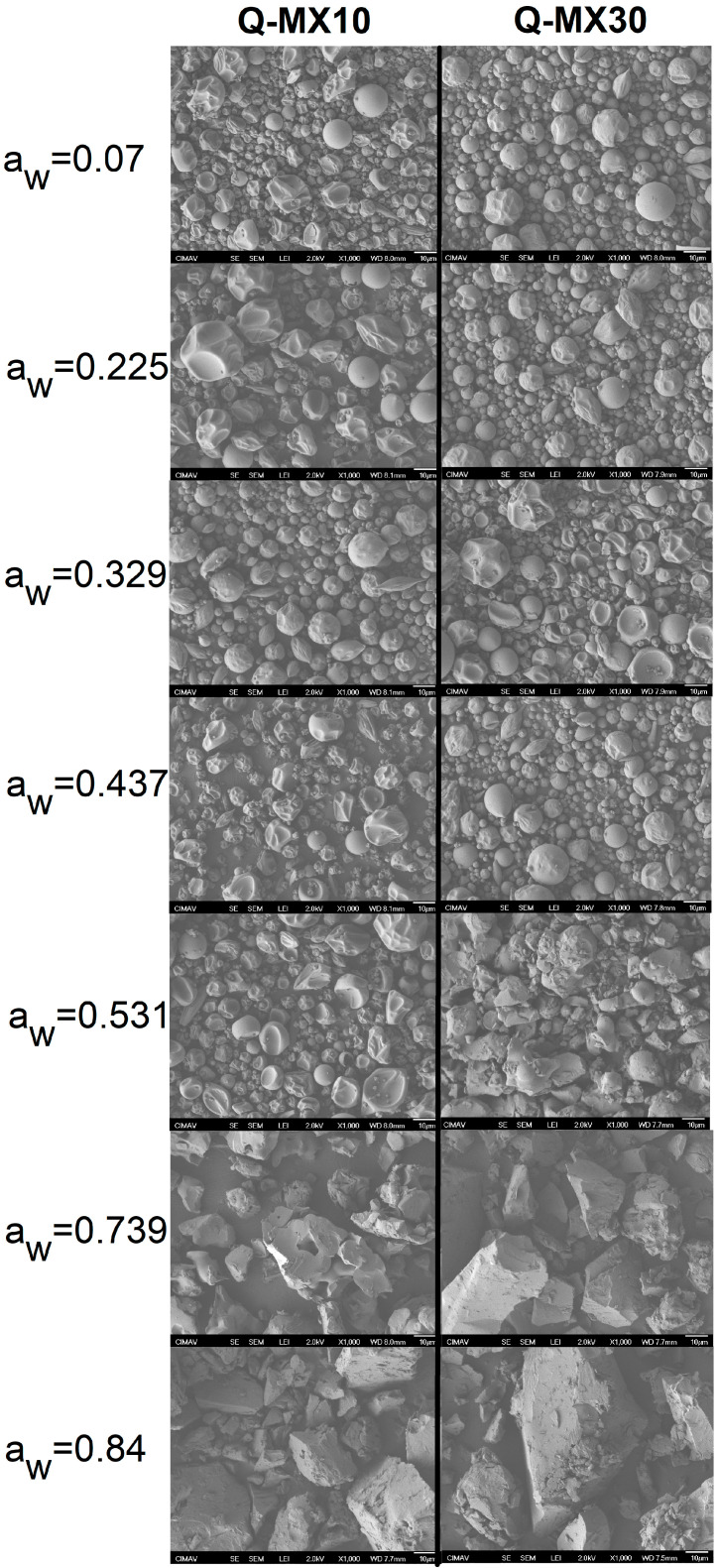
Scanning electron microscopy (SEM) images of samples Q-MX10 and Q-MX30 at 1000× magnification, illustrating the morphological changes under different storage humidity conditions at 25 °C.

The morphological analysis performed using SEM is shown in Figure 3. This figure presents a representative micrograph acquired at 1000X magnification of samples Q-MX10 and Q-MX30 subjected to various storage humidity conditions at 25 °C.

For both samples within the a_w_ range of 0.070–0.329, a very similar behavior was observed, exhibiting a comparable morphology with particle sizes ranging between 2 and 15 μm. The observed morphologies included semi-spherical shapes with smooth surfaces as well as particles with irregular (rough) surfaces. As the water activity increased (a_w_ = 0.437), a change was noticed in some particles within the samples, displaying irregular spheroidal structures with the appearance of deflated balloons; this phenomenon is associated with the limit of moisture adsorption stability. At an a_w_ of 0.531, the samples exhibited a different morphology, resembling clusters or agglomerates of irregularly shaped spherical particles larger than 15 µm. Finally, at water activities higher than 0.739, crystallized structures with irregular shapes and smooth surfaces were observed. These observations suggest that the adsorbed water in the samples induced crystallization, leading to changes in particle morphology and size, resulting in a rigid, crystalline solid material. The SEM analysis of MD10 and MD30 provided insights into the morphology of spray-dried samples and established the stability threshold at which microstructural changes occur due to moisture adsorption, altering the properties of the powders. Navarro-Flores et al. [47] conducted a study on the spray drying of a native plant rich in phenolic compounds using maltodextrin as a carrier agent. They reported that the resulting microcapsules exhibited irregular shapes, smooth surfaces with depressions, and particle sizes ranging from 3 to 8 µm. Ferrari et al. [48] reported on the stability of spray-dried blackberry powder using maltodextrin (DE of 20) and gum Arabic as carrier agents. The particles exhibited spherical shapes of various sizes. Specifically, for the samples obtained using maltodextrin, the microcapsules were found to have smooth and wrinkled surfaces, with a size of 43.67 ± 1.76 µm. Additionally, it was noted that during storage for 150 days at 25 and 35 °C, the powders showed a significant tendency to agglomerate.

**Figure 4:**
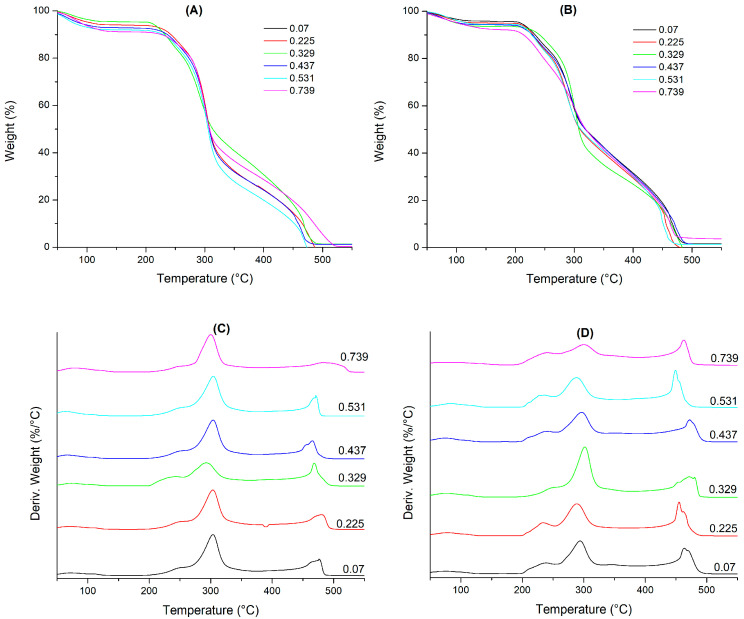
TGA results for spray-dried samples under various humidity adsorption conditions at 25 °C: (**A**,**C**) Q-MX10; (**B**,**D**) Q-MX30.

Thermogravimetric analysis (TGA) quantifies mass loss in relation to temperature increase. Figure 4A,B present the TGA results for the spray-dried Q-MX10 and Q-MX30 samples subjected to different humidity adsorption conditions at 25 °C. Figure 4C,D display the derivative weight curves relative to temperature, providing a clearer view of the onset and completion of each thermal event. In general, four thermal events were identified for both types of maltodextrins, observed under all humidity adsorption conditions: the first event occurs within a temperature range of 50–150 °C, corresponding to an 8% mass loss due to water evaporation; the second thermal event, occurring between 200 and 250 °C, involves a mass loss of approximately 40%, likely related to the degradation of low-molecular-weight carbohydrates; the third event, observed between 250 and 325 °C, is associated with a 40% mass loss, attributed to the thermal degradation of high-molecular-weight carbohydrates; finally, the fourth thermal event appears between 425 and 500 °C, with a 10% mass loss corresponding to the calcination or final degradation of the organic matter in the sample. Costa Ferreira et al. [49] microencapsulated phenolic compounds from the by-product of tucum almonds (*Astrocaryum vulgare* Mart.) using maltodextrin as a carrier agent in spray drying. Their TGA analysis revealed only three thermal events: the first in the temperature range of 50–100 °C, the second between 200 and 239 °C, and the third from 283 to 356 °C. On the other hand, Saavedra-Leos et al. [25] reported a well-defined thermal event at approximately 115 °C, which was observed only in samples with the highest a_w_ values (i.e., a_w_ = 0.75). This event was attributed to thermal hydrolysis induced by the large amount of adsorbed water and supplied heat. However, this thermal event was not observed in the TGA analysis presented in this study. In sugar polymers, thermal degradation induces caramelization (Maillard reaction), generating aromas, color changes (yellow and brown tones), and flavor shifts from smooth, caramelized sweetness to bitter, burnt notes. The thermal degradation process involves stages such as enolization, dehydration, carbonyl cleavage, dicarbonyl scission, retroaldolization, aldolization, and radical reactions [50].

**Figure 5:**
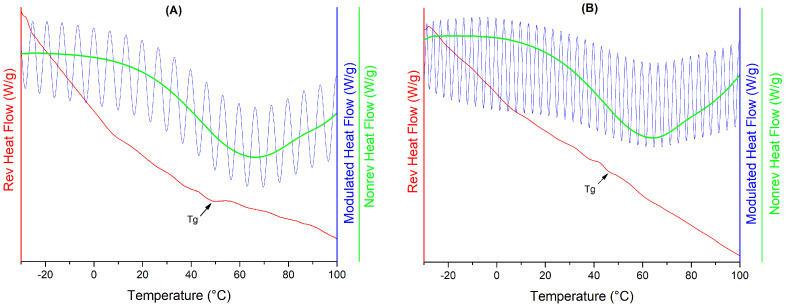
MDSC thermograms for samples at an a_w_ of 0.07: Q-MX10 (**A**), and Q-MX30 (**B**).

Figure 5 shows the MDSC thermograms for samples Q-MX10 and Q-MX30 at an a_w_ of 0.07. Each graph displays three curves: the reversible heat flow (left axis, in red), the modulated heat flow (first right axis, in blue), and the non-reversible heat flow (second right axis, in green). The glass transition temperature (Tg) is observed in the reversible heat flow curve, while other thermal events, such as melting and degradation, are observed in the non-reversible heat flow curve [29]. The Tg appears as a smooth change in the slope of the curve, indicated by arrows in the figures. Table 2 summarizes the Tg values for samples Q-MX10 and Q-MX30 under different humidity conditions. Although both samples exhibited similar behavior, with Tg decreasing monotonically with increasing a_w_, sample Q-MX30 presented slightly lower values. The Tg varied from 44.2 to −10.99 °C for sample Q-MX10 and from 44.2 to −9.5 °C for sample Q-MX30. This is a common behavior observed in carbohydrate polymers like maltodextrins, where water molecules act as plasticizers [51]. They increase the free volume between glucose molecules, promoting the transition of the microstructure from a glassy state to a rubbery state.

**Figure 6:**
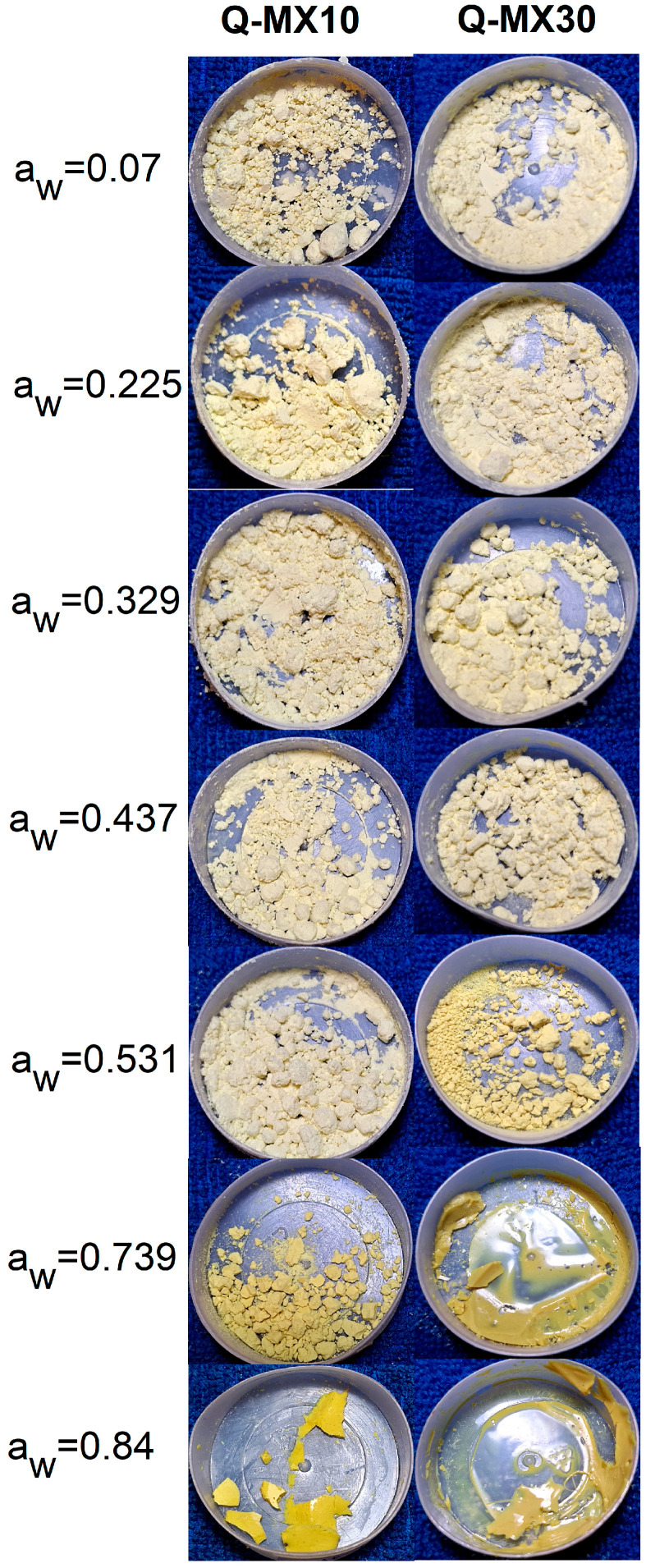
Optical photographs of the Q-MX10 and Q-MX30 samples subjected to different humidity conditions at 25 °C.

The general appearance of the powders was studied using optical microscopy. Figure 6 shows optical photographs of the Q-MX10 and Q-MX30 samples subjected to different humidity conditions. Both samples exhibited similar behavior, differing only in the water activity level at which the observed changes occurred. Initially, for a_w_ values of 0.07–0.531, the Q-MX10 sample appeared as a dry, fine, non-agglomerated powder with a light-yellow color. The Q-MX30 sample showed the same appearance in the a_w_ range of 0.07–0.437. At an a_w_ of 0.739 for sample Q-MX10, and a_w_ of 0.531 for sample Q-MX30, a change to a more intense yellow color and increased particle agglomeration was observed. As a_w_ increased further, the powder crystallized due to moisture adsorption, resulting in a continuous, rigid material with a shiny appearance and intense yellow color. For sample Q-MX10, this occurred at an a_w_ of 0.739, whereas for sample Q-MX30, it was observed at an a_w_ of 0.531. Evidently, the differences in water activity observed here are caused by the differences in DE between the two maltodextrins, with the Q-MX30 sample tending to adsorb more moisture than the Q-MX10 sample. Saavedra-Leos et al. [25] observed the macroscopic behavior of a set of four maltodextrins with DE ranging from 10 to 40 using optical photography. They reported a color change in the powder from white to pale yellow up to an a_w_ range of 0.434–0.532. Beyond this a_w_ value, the maltodextrins exhibited various physical changes, such as particle agglomeration, volume shrinkage, the appearance of a rubbery state, and moisture saturation. These changes were more pronounced for maltodextrins with higher DE values.

### 3.3. Antioxidant Activity of the Functional Food

**Figure 7:**
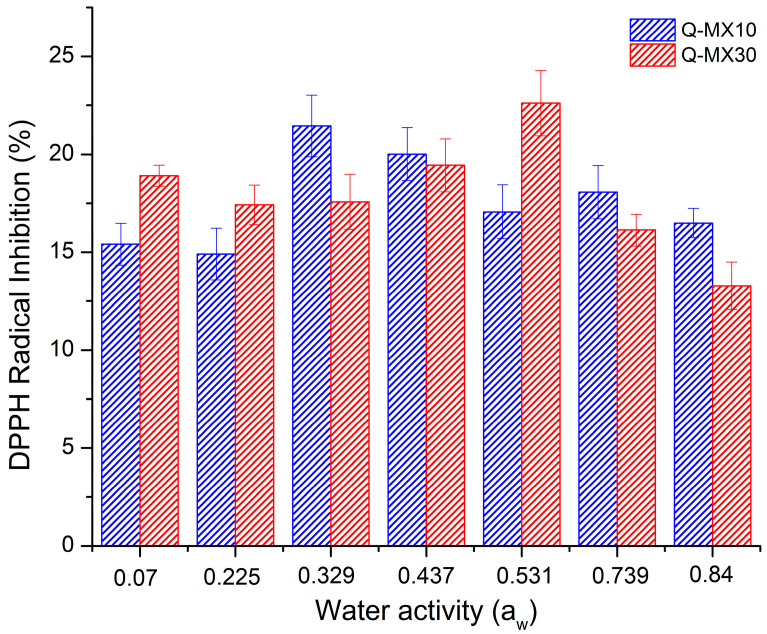
Antioxidant activity, expressed as the percentage inhibition (%) of DPPH radicals, for Q-MX10 and Q-MX30 samples under various moisture adsorption conditions at 25 °C. The values represent the average of three measurements.

The determination of antioxidant activity (AA) was carried out by scavenging free radicals using the DPPH reagent as a standard. Figure 7 shows the results for the AA of Q-MX10 and Q-MX30 samples subjected to different moisture adsorption conditions at 25 °C. AA is expressed as the percentage inhibition (%) of DPPH radicals due to hydrogen donation for the neutralization of free radicals (OH-). For sample Q-MX10 after the drying process, the initial AA value was 18.02 ± 2.03%, while for sample Q-MX30, the initial AA value was 22.73 ± 1.65%. In order to evaluate the effect of storage conditions (water activity) and the type of carrier agent (DE 10 and DE 30) on the AA value, an ANOVA analysis was conducted with a significance level of α = 0.05. The results indicated that there was no significant difference in the mean AA value across the different storage conditions. Additionally, there was no significant effect of using MX10 or MX30 on the AA. This indicates that under these storage conditions, the microencapsulated antioxidant compound in the maltodextrin is preserved, preventing degradation due to the absorbed water. Georgetti et al. [52] evaluated the chemical and biological properties of spray-dried soy extract employing three carrying agents. The results indicated an AA of 59% for concentrated soy extract, 55% for silicon dioxide, 52% for maltodextrin, and 50% for starch. Recently, the antioxidant activity of quercetin microencapsulated in inulin, maltodextrin, and their blends was compared. It was found that in the case of maltodextrin, quercetin exhibits greater interaction, which reduces its bioactivity in interacting with free radicals. As a result, the AA is lower with maltodextrin compared to inulin [32]. For a 25–75% maltodextrin–inulin blend containing quercetin and *B. claussi* as a probiotic, the AA in the a_w_ range of 0.073–0.856 was statistically similar. This suggests that the humidity conditions did not significantly impact the antioxidant’s preservation capacity [33].

## 4. Discussion

### Equilibrium State Diagram

**Figure 8:**
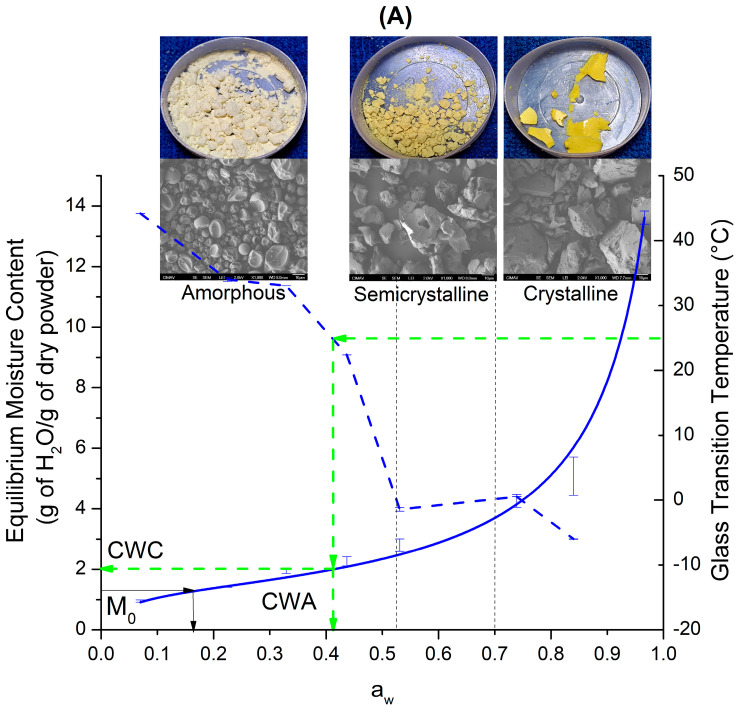

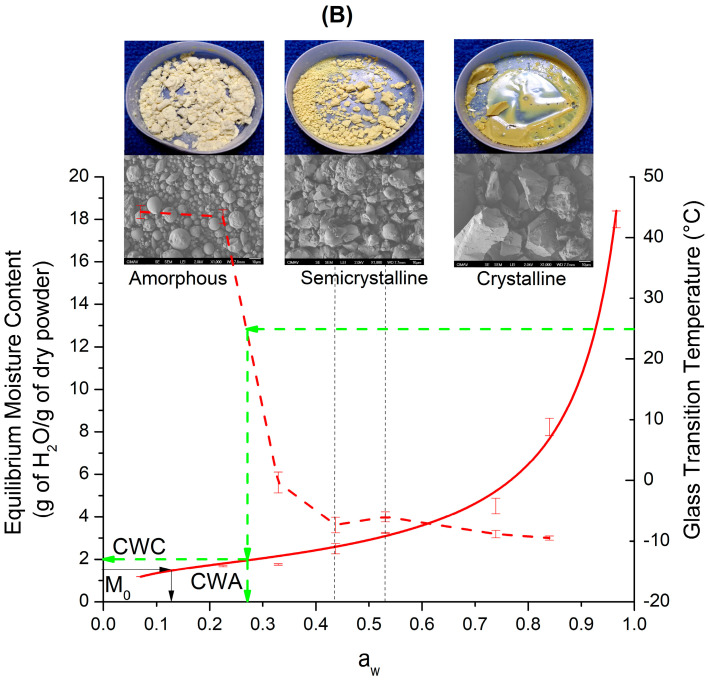
Equilibrium state diagram for samples Q-MX10 (**A**) and Q-MX30 (**B**), constructed from the adsorption isotherms at 25 °C, and the physicochemical characterization results. In both figures, the continuous lines (blue and red) represent the adsorption isotherm, the dotted lines (blue and red) represent the Tg curve, and the dotted green lines the critical storage conditions.

Equilibrium state diagrams can be constructed by combining water sorption isotherms and Tg to determine the critical values for water content and water activity at a specific storage temperature [53]. However, these diagrams can provide additional physicochemical information that helps to understand the behavior of the powder during storage. In this context, Figure 8 presents the equilibrium state diagram for samples Q-MX10 and Q-MX30, constructed based on the physicochemical characterization results. The diagram’s main feature includes the adsorption isotherms at 25 °C described by the GAB model, depicted as continuous blue and red lines for samples Q-MX10 and Q-MX30, respectively, which are read on the left *Y*-axis. By comparing the two isotherms, the state diagram reveals that while the adsorption behavior of both maltodextrins is similar in shape, there is a slight difference in the equilibrium moisture content adsorbed. Specifically, sample Q-MX30 adsorbs more water than sample Q-MX10. This behavior is evidently related to the differences in the DE between the two maltodextrins. Additionally, the Tg data are displayed as dashed lines in blue (Q-MX10) and red (Q-MX30), corresponding to the right *Y*-axis. Furthermore, to the individual information obtained from the isotherm and Tg (as previously discussed), combining these data provides parameters related to storage conditions, such as the critical water content (CWC), the critical water activity (CWA), and the amount of moisture adsorbed at a given storage temperature. The CWC and CWA are important thresholds that indicate the limits within which a material can be stored safely without compromising its integrity. Critical parameters can be extrapolated from the intersection of the storage temperature line (dashed green line) with the Tg curve and subsequently with the isotherm curve. These values are read directly from their respective axes. At the storage temperature of 25 °C, the CWC values are nearly identical for both samples, at 1.98 g of H_2_O per g of dry powder. However, the CWA values are slightly different, with 0.41 for sample Q-MX10 and 0.27 for sample Q-MX30. In the state diagram is indicated the calculated monolayer water content (M_0_) for each isotherm, which were 1.2 and 1.5 for samples Q-MX10 and Q-MX30, respectively. When comparing the M_0_ values against the CWC, the latter is several times higher. M_0_ represents the amount of water adsorbed in a single molecular layer on the surface of the material, it is often considered the most stable state for a material because it indicates that all available binding sites for water on the surface are occupied, and any additional water will lead to multilayer adsorption, which can be less stable. On the other hand, CWC indicates the water content at which a material transitions from a glassy (solid, stable) state to a rubbery (less stable, more reactive) state. It represents the maximum water content at which the material can maintain its structural integrity and stability [11,54,55]. When the CWC is significantly higher than the M_0_, it means the material can tolerate a relatively high amount of water before becoming unstable. This suggests good moisture resistance and a stable glassy state over a wider range of water content.

In addition to the aforementioned data, the equilibrium state diagram provides further insights into the microstructure, morphology, and appearance of the samples. This information is represented by vertical dotted lines, indicating the thresholds at which microstructural transitions occur. These lines help visualize and understand the changes in the material’s physical properties under different conditions. Based on the physicochemical characterization results from XRD, SEM, and optical microscopy, three distinct transition regions were identified: amorphous, semicrystalline, and crystalline. In the amorphous region (a_w_ range: 0.07–0.437), the microparticles exhibit a predominantly spherical morphology. The overall appearance is a fluffy powder with a pale-yellow color. The corresponding Tg values range from 44 to −7 °C. The semicrystalline region (a_w_ range: 0.437–0.739), or intermediate region, shows a semicrystalline behavior. Here, very-low-intensity diffraction peaks are observed above the broad peak in the XRD pattern. The particles begin to collapse, displaying merged spherical particles with irregular shapes. The overall appearance is an agglomerated powder with an intense yellow color. The Tg values in this region are practically below 2 °C. In the crystalline region (a_w_ above 0.739) the microstructure of the functional food is completely collapsed. The morphology of the particles becomes fully irregular, and the overall appearance is that of a continuous solid material with an intense yellow color. These transitions help us to understand the material’s stability and behavior under varying humidity conditions.

Finally, the optimal storage conditions for these functional foods, which are based on quercetin microencapsulated in maltodextrins with different dextrose equivalents, lie within the amorphous region. These conditions are defined by the CWC and CWA values. The ideal storage parameters are a temperature of 25 °C, an a_w_ of 0.437, and a maximum water content of 1.98 g of H_2_O per gram of dry powder. While these functional foods can tolerate storage temperatures up to 44 °C, the maximum water content is constrained by the monolayer water content value, which is 1.2 g of H_2_O per gram of dry powder. In various studies where maltodextrin is used as a microencapsulating agent, storage temperatures close to 50 °C have been reported. However, to prevent microstructural changes, the moisture content at these temperatures must be kept below 0.1 g of H_2_O per gram of dry powder. Clearly, in such cases, very strict humidity control is required to avoid the collapse of the microstructure [56,57]. 

## 5. Conclusions

This study investigates the equilibrium state diagram of functional foods based on maltodextrins (MX) with varying dextrose equivalents (DE 10 and DE 30) for quercetin microencapsulation via spray drying. Equilibrium adsorption isotherms at 25 °C were constructed to compare the adsorption behavior of these polymeric foods. Among the adsorption models tested, the GAB model presented better fitting results than the BET model. Both samples exhibited similar monolayer water content (M_0_) of approximately 1.2–1.5 g of water per g of dry powder. Physicochemical characterization techniques, including XRD, SEM, TGA, MDSC, and optical microscopy, provided comprehensive insights into the microstructural transitions occurring under varying humidity conditions. An equilibrium state diagram was constructed to predict the optimal storage conditions for the functional foods. Three transition regions—amorphous, semicrystalline, and crystalline—were identified based on differences in crystallinity through XRD analysis, changes in particle morphology observed in SEM micrographs, and variations in powder appearance and color noted from optical microscopy. The optimal storage conditions were extrapolated from the isotherms and Tg curves, defined by the CWC and CWA thresholds: a temperature of 25 °C, an a_w_ of 0.437, and a maximum water content of 1.98 g of water per gram of dry powder. The antioxidant activity showed similar values despite varying water content, indicating the preservation of the antioxidant within the carrying agent across the complete range of water activities. This research enhances understanding of maltodextrin behavior across diverse dextrose equivalents, aiding in the development of stable encapsulated products.

## Figures and Tables

**Table 1:** Mathematical models used to describe sorption behavior of dry food products.

Model Name	Model and Describing Parameters
GAB	$\mathrm{Xe}=\frac{{\mathrm{XmCKa}}_{w}}{\left(1-{\mathrm{Ka}}_{w}\right)\left(1-{\mathrm{Ka}}_{w}+{\mathrm{CKa}}_{w}\right)}$ X_e_ = equilibrium water content (g H_2_O/g dry mass) X_m_ = equilibrium monolayer water content (g H_2_O/g dry mass) a_w_ = water activity K and C = model constants
BET	$\mathrm{Xe}=\frac{{\mathrm{XmCa}}_{w}}{\left(1-a_{w}\right)\left(1+\left(C-1\right)a_{w}\right)}$ Xe = equilibrium water content (g H_2_O/g dry mass) X_m_ = equilibrium monolayer water content (g H_2_O/g dry mass) a_w_ = water activity C = model constant

**Table 2:** Tg values of samples Q-MX10 and Q-MX30 under different humidity conditions.

Water Activity (a_w_)	Sample	
	Q-MX10 Tg (°C)	Q-MX30 Tg (°C)
0.07	44.2 ± 0.04	44.22 ± 0.01
0.225	33.98 ± 0.02	43.43 ± 0.03
0.329	33.05 ± 0.07	−0.34 ± 0.03
0.437	2.89 ± 0.15	−7.35 ± 0.24
0.531	−9.16 ± 0.2	−6 ± 0.02
0.739	−11.2 ± 0.21	−8.83 ± 0.36
0.84	−10.99 ± 0.63	−9.5 ± 0.4

## Data Availability

The original contributions presented in the study are included in the article. Further inquiries can be directed to the corresponding author/s.
